# Recent advances in medical therapy for metastatic urothelial cancer

**DOI:** 10.1007/s10147-018-1260-0

**Published:** 2018-03-20

**Authors:** Takeshi Yuasa, Shinji Urakami, Junji Yonese

**Affiliations:** 1 Department of Urology, Cancer Institute Hospital, Japanese Foundation for Cancer Research, Ariake, Tokyo 135-8550 Japan; 2 Department of Urology, Toranomon Hospital, Tokyo, Japan

**Keywords:** Immune-checkpoint inhibitor, Pembrolizumab, GC regimen, MVAC regimen, Urothelial cancer, Chemotherapy

## Abstract

Cytotoxic chemotherapy has been the mainstay of medical therapy for metastatic urothelial cancer. Currently, the gemcitabine/cisplatin regimen is widely used worldwide as the standard first-line medical treatment. Very recently, in 2017, pembrolizumab, a highly selective, humanized monoclonal IgG4κ isotype antibody against programmed death 1, was approved as a second-line treatment to be used after platina-based chemotherapy for metastatic urothelial cancer in Japan. Based on its promising anti-tumor efficacy and manageable safety profile as demonstrated in the phase III KEYNOTE-045 trial, pembrolizumab therapy is expected to be rapidly introduced for treating metastatic urothelial cancer in clinical practice. The paradigm of medical treatment for patients with metastatic UC is dramatically changing through the introduction of this and other immune-checkpoint inhibitors. In this article, we provide a brief overview of these immune-checkpoint inhibitors and a comprehensive summary of the use of cytotoxic chemotherapy for metastatic urothelial cancer, including ongoing clinical trials.

## Introduction

Cytotoxic chemotherapy has been the mainstay of medical therapy for patients with metastatic urothelial cancer (UC) for a long time. Since 1985, when Sternberg et al. reported the excellent results of the cisplatin-based multi-agent chemotherapy regimen known as MVAC (methotrexate, vinblastine, adriamycin, cisplatin), no medical treatment has been more effective [1]. Only one clinical trial comparing a gemcitabine-plus-cisplatin regimen (GC) with MVAC demonstrated that the GC regimen had a treatment efficacy similar to that of MVAC while causing less toxicity than MVAC [2, 3]. Currently, therefore, the GC regimen is widely used worldwide as the standard first-line medical treatment.

On December 25, 2017, pembrolizumab (Keytruda^®^, Merck), a highly selective, humanized monoclonal IgG4κ isotype antibody against programmed death 1 (PD-1) that selectively inhibits the interaction between PD-1 (which is expressed on activated T cells) and PD-1 ligand 1 (PD-L1) and 2 (PD-L2) [which are expressed on antigen-presenting cells (APC) and cancer cells] was approved as a second-line treatment for use after platina-based chemotherapy for patients with metastatic UC in Japan.

We are currently on the verge of the second breakthrough in the medical treatment of metastatic UC since the discovery of MVAC therapy. The paradigm of medical treatment for metastatic UC is dramatically changing through the introduction of this and other immune-checkpoint inhibitors. The United States Food and Drug Administration (US-FDA) has approved five immune-checkpoint inhibitors including pembrolizumab, atezolizumab (Tecentriq^®^, Roche), nivolumab (Opdivo^®^, Ono/Bristol-Myers\Squibb), avelumab (Bavencio^®^, Merck, Pfizer, Eli Lilly), and durvalumab (Imfinzi^®^, Medimmune/AstraZeneca). Various clinical trials currently underway are attempting to increase the efficacy of each of these checkpoint inhibitors by combining them with other immunogenic agents and with cytotoxic chemotherapy. In this article, we provide a brief overview of these immune-checkpoint inhibitors and a summary of comprehensive medical treatment using cytotoxic chemotherapy for metastatic UC, including ongoing clinical trials. The doses and schedules of the regimens currently administered in Japan are shown in Table 1.

**Table 1:** Doses and schedules of current regimens for metastatic UC in Japan

Drug	Dose and schedule	Duration of cycles (days)
Pembrolizumab		
Pembrolizumab	200 mg, day 1	21
Gemcitabine/Cisplatin (GC) regimen		
Gemcitabine	1 g/m^2^, days 1, 8, 15	28
Cisplatin	70 mg/m^2^, day 2	
MVAC regimen		
Methotrexate	30 mg/m^2^, days 1, 15, 22	28
Vinblastine	3 mg/m^2^, days 2, 15, 22	
Adriamycin	30 mg/m^2^, day 2	
Cisplatin	70 mg/m^2^, day 2	
Gemcitabine/Carboplatin (GCa) regimen		
Gemcitabine	1 g/m^2^, days 1, 8	21
Carboplatin	AUC5, day 2	
Paclitaxel**/**cisplatin**/**gemcitabin (PCG) regimen		
Paclitaxel	80 mg/m^2^, days 1, 8	21
Cisplatin	50–70 mg/m^2^, days 2	
Gemcitabine	1 g/m^2^, days 1, 8	
Gemcitabine and paclitaxel (GP) regimen		
Gemcitabine	1 g/m^2^, days 1, 8	21
Paclitaxel	175 mg, day 1	

*AUC* area under the concentration curve

### Immune-checkpoint inhibitors

**Fig. 1:**
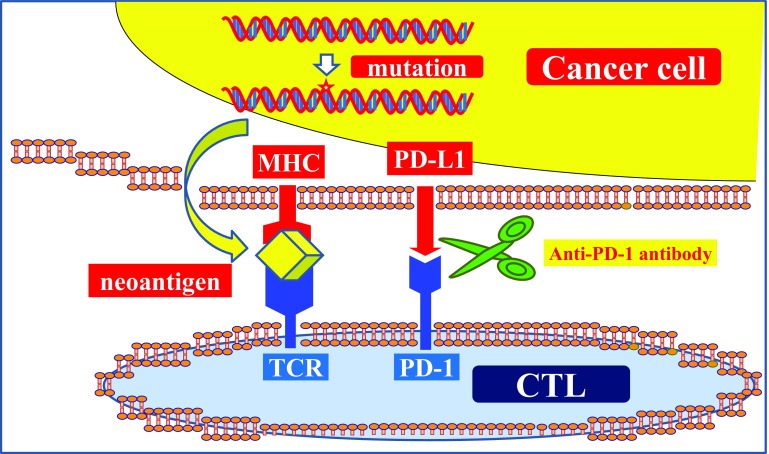
Anti-PD-1/PD-L1 novel immune therapy. When a complex of PD-L1 expressed by cancer cells engages PD-1 expressed on CD8-positive CTLs, immune tolerance is achieved. Destruction of this immune tolerance using immune-checkpoint inhibitors is the latest novel form of immune therapy. *MHC* major histocompatibility, *CTL* cytotoxic T lymphocytes, *PD-1* programmed death-1, *PD-L1* programmed death ligand 1, *TCR* T cell receptor

PD-L1 and PD-L2, which are known to be expressed on the surfaces of APC and cancer cells, engage PD-1, which is expressed on CD8-positive cytotoxic T lymphocytes (CTLs), as negative immune regulators [4, 5]. When the complex of PD-1 and PD-L1/PD-L2 is formed, immune tolerance is achieved. Novel immune therapy using immune-checkpoint inhibitors can destroy this immune tolerance (Fig. 1). When immune tolerance is broken by immune-checkpoint inhibitors, the CD8-positive CTLs can recognize the neoantigens from cancer cells that are presented on major histocompatibility class I (MHC-I) or class II (MHC-II) molecules. Thus, they are activated and proliferate, leading to an antigen-specific immune response that kills neoantigen-bearing cancer cells [4, 5]. This is the functional mechanism of this novel immune therapy (Fig. 1). In addition, the main results of the clinical trials using current immune-checkpoint inhibitors for the patients with metastatic UC are shown in Table 2.

**Table 2:** Main results of clinical trials using current immune-checkpoint inhibitors for the patients with metastatic urothelial cancer

Agent	Trading name	Antibody	Dose and schedule	Phase	Trial name	Patients number	Main results	Reference
Pembrolizumab	Keytruda	PD-1	200 mg, every 3 weeks	3	Keynote-045	542 (270 versus 272)	The median OS of pembrolizumab arm (10.3 months) was significantly longer than that of the chemotherapy arm (7.4 months)	[6]
Nivolumab	Opdivo	PD-1	3 mg/kg, every 2 weeks	2	Checkmate 275	270	Confirmed ORR for all patients was 19.6%. Severe AEs (grade 3–4) occurred in 18% of patients	[9]
Atezolizumab	Tecentriq	PD-L1	1200 mg, every 3 weeks	3	IMvigor211	931 (467 versus 464)	The median OS of the pre-stratified PD-L1 high expression patients (> 5%) did not differ significantly between the atezolizumab arm (11.1 months) and the chemotherapy arm (10.6 months)	[10]
Durvalumab	Imfinzi	PD-L1	10 mg/kg, every 2 weeks	1/2		191	The overall ORR was 17.8% including 7% CR. Severe AEs (grade 3–4) and grade 3–4 immune-mediated AEs occurred in 6.8 and 2.1% of the patients, respectively	[11]
Avelumab	Bavencio	PD-L1	10 mg/kg, every 2 weeks	1	JAVELIN	161	The ORR was 17% including 6% with CR. Severe (grade 3–4) treatment-related AEs occurred in 8%	[12]

*PD-1* programmed death 1*, PD-L1* programmed death ligand 1, *OS* overall survival, *ORR* objective response rate, *CR* complete response, *AE* adverse event, *Reference* the reference number in this review article

### Pembrolizumab

The essential clinical trial of pembrolizumab for metastatic UC is the KEYNOTE-045 study [6]. The KEYNOTE-045 study is an open-label, international, phase III clinical trial that randomly assigned 542 patients with advanced UC that had recurred or progressed after platinum-based chemotherapy to receive either pembrolizumab at a dose of 200 mg or the investigator’s choice of chemotherapy with paclitaxel, docetaxel, or vinflunine every 3 weeks [6]. In this study, pembrolizumab was associated with significantly longer overall survival (OS) and a lower rate of adverse events (AEs) compared to chemotherapy as the second-line therapy for platinum-refractory advanced UC [6]. The median OS in the pembrolizumab-treated patients was 10.3 months [95% confidence interval (CI) 8.0–11.8 months] [6]. The median OS in the chemotherapy-treated patients, on the other hand, was 7.4 months (95% CI 6.1–8.3 months). This difference was significant [hazard ratio (HR) 0.73, 95% CI 0.59–0.91, *P *= 0.002] [6]. In addition, among patients who had high PD-L1 expression (10% or more), the median OS was 8.0 months for those treated with pembrolizumab (95% CI 5.0–12.3 months) as compared to 5.2 months for those treated with chemotherapy (95% CI 4.0–7.4 months, HR, 0.57, 95% CI 0.37–0.88, *P *= 0.005) [6]. There was no significant difference in progression-free survival (PFS) period either among all treated patients or among high PD-L1 expression patients [6]. As for AEs, pembrolizumab-treated patients experienced significantly fewer events of any grade and significantly fewer events of grades 3, 4, and 5 (60.9 and 15.0%, respectively) compared to chemotherapy-treated patients (90.2 and 49.4%, respectively) [6].

Pembrolizumab has received accelerated approval from the US-FDA as a first-line therapy for patients with cisplatin-containing chemotherapy-ineligible metastatic UC. The KEYNOTE-052 study is an ongoing single-arm, open-label trial for patients with locally advanced or metastatic UC who are ineligible for cisplatin-containing chemotherapy [7]. This study contains 370 patients. At a median follow-up of 7.8 months, the objective response rate (ORR) was approximately 29% and the median duration of response had not yet been reached. The most common side effects in the two completed trials included fatigue, musculoskeletal pain, decreased appetite, nausea, and diarrhea [6, 7].

Currently, a multi-center international randomized clinical trial including data from Japan is comparing pembrolizumab monotherapy and standard chemotherapy with or without pembrolizumab as first-line therapy for patients with previously untreated advanced UC.

### Other checkpoint inhibitors

To date, as described above, five checkpoint inhibitors including pembrolizumab have been shown to be efficacious and have accordingly received approval from the US-FDA for metastatic UC. Here, we provide a brief overview of these other agents, although they have not yet been approved in Japan.

### Nivolumab

Nivolumab is a fully human IgG4 anti-PD-1 monoclonal antibody similar to pembrolizumab. After its promising anti-tumor efficacy against metastatic renal cell cancer (RCC) and its manageable safety profile were demonstrated in the phase III Checkmate025 trial, nivolumab was launched in 2016 and has been rapidly introduced in clinical practice for metastatic RCC in Japan [8].

For patients with metastatic UC who were previously treated with at least one platinum-based chemotherapy regimen, the Checkmate275 international, multi-institutional, phase II, single-arm study was conducted [9]. In this trial, 270 patients from 63 sites in 11 countries received nivolumab, and 265 were evaluated for activity [9]. At a median follow-up of 7 months, median OS was 8.7 months (95% CI 6.1 months to not reached) among all patients, 11.3 months (95% CI 8.7 months to not reached) in patients with high PD-L1 expression (≥ 1%), and 5.9 months (95% CI 4.30–8.08 months) in those with low PD-L1 expression (< 1%) [9]. Confirmed ORR for all patients was 19.6% (95% CI 15.0–24.9) [9]. In addition, nivolumab produced a response in 28.4, 23.8, and 16.1% of patients with PD-L1 expression levels ≥ 5, ≥ 1, and < 1%, respectively [9]. Regarding AEs, 64% of patients experienced an AE of any grade; the most common of these was fatigue (17%) [9]. Severe AEs (grades 3–4) occurred in 18% of patients; the most common grade 3 AEs were fatigue and diarrhea (2%) [9].

Nivolumab is currently being tested in two clinical trials for advanced UC in Japan. The first study is a phase II single-arm study for patients with advanced UC who were previously treated with at least one platinum-based chemotherapy regimen. The other is a phase III international, multi-institutional clinical trial comparing the combination of nivolumab and ipilimumab (Yervoy^®^, Bristol-Myers Squibb), a checkpoint inhibitor of another type that blocks the complex of CTL-associated protein 4 (CTLA-4) and its ligand, with the combination of nivolumab and standard chemotherapy, and standard chemotherapy as first-line therapy for previously untreated patients.

### Atezolizumab

Although atezolizumab was the first agent to receive approval from the US-FDA for advanced UC, it failed to demonstrate superiority to chemotherapy as a second-line therapy in a large phase III trial [10]. Atezolizumab is a fully humanized, engineered monoclonal IgG1 antibody against PD-L1. A multi-institutional, open-label, phase III randomized controlled trial (IMvigor211) for patients (*n* = 931) with advanced UC who had progressed after platinum-based chemotherapy was conducted to compare the efficacy of atezolizumab 1200 mg (*n* = 467) with that of chemotherapy of the physician’s choice (*n* = 464, vinflunine 320 mg/m^2^, paclitaxel 175 mg/m^2^, or docetaxel 75 mg/m^2^) [10]. Randomization was stratified by PD-L1 expression {categories were < 1% [immunohistochemistry (IC0)] or 1% to < 5% (IC1) of tumor-infiltrating immune cells versus ≥ 5% of tumor-infiltrating immune cells (IC2/3)} [10]. Although the primary endpoint of OS was tested hierarchically in pre-specified IC2/3 populations (*n* = 234), OS did not differ significantly between the atezolizumab group and the chemotherapy group (median 11.1 months, 95% CI 8.6–15.5 versus 10.6 months, 95% CI 8.4–12.2, *P *= 0.41) [10]. Confirmed ORRs were also similar between treatment groups in the IC2/3 population: 23 and 22% in the atezolizumab and chemotherapy groups, respectively [10]. In the intention-to-treat population, patients receiving atezolizumab had fewer grade 3–4 treatment-related AEs than did those receiving chemotherapy (20 versus 43%) as well as fewer AEs leading to treatment discontinuation (7 versus 18%) [10].

Atezolizumab is currently being tested in a phase III international, multi-institutional clinical trial comparing atezolizumab monotherapy, a combination of atezolizumab and standard chemotherapy, and standard chemotherapy as first-line therapy for patients with previously untreated advanced UC.

### Durvalumab

Durvalumab is a human IgG1 kappa monoclonal antibody against PD-L1. Durvalumab (10 mg/kg every 2 weeks) was tested in a phase I/II open-label study (*n* = 191) for patients with advanced UC who had experienced disease progression during, were ineligible for, or had refused chemotherapy [11]. The overall ORR was 17.8% (95% CI 12.7–24.0%), including 7% complete responses (CR). ORRs were 27.6% (95% CI 19.0–37.5%) and 5.1% (95% CI 1.4–12.5%) in patients with high and low or negative expression of PD-L1, respectively [11]. Median PFS and OS periods were 1.5 months (95% CI 1.4–1.9 months) and 18.2 months (95% CI 8.1 months to not estimable), respectively [11]. Severe AEs (grade 3–4) and grade 3–4 immune-mediated AEs occurred in 6.8 and 2.1% of the patients, respectively [11].

Durvalumab is currently being tested in a phase III international, multi-institutional clinical trial to compare durvalumab monotherapy, a combination of durvalumab and tremelimumab (AstraZeneca), a fully human monoclonal antibody against anti-CTLA-4, and standard chemotherapy as first-line therapy for patients with previously untreated advanced UC.

### Avelumab

Avelumab is a fully human monoclonal antibody against PD-L1. In a phase I study for post-platinum patients with at least 6 months of follow-up (*n* = 161), the ORR was 17% (95% CI 11–24%), including 6% with CR [12]. The most frequent AEs (of any grade in ≥ 10% patients) were infusion-related reaction (29%, all grade 1–2) and fatigue (16%). Severe (grade 3–4) treatment-related AEs occurred in 8%; the most common of these were fatigue (2%) and asthenia, elevated lipase, hypophosphatemia, and pneumonitis (1%) [12]. It is worth remembering that avelumab causes infusion-related reactions frequently, whereas this is seldom seen in the other checkpoint inhibitors.

### Cytotoxic chemotherapy

Although these immune-checkpoint inhibitors have led to breakthroughs in medical therapy for patients with metastatic UC, cytotoxic chemotherapy is still the standard first-line therapy. Among the chemotherapeutic regimens, the GC regimen is the best known and most frequently used regimen.

### Gemcitabine and cisplatin (GC) regimen

As the GC regimen, consisting of gemcitabine 1000 mg/m^2^ on days 1, 8, and 15 and cisplatin 70 mg/m^2^ on day 2 (Table 1), provides a survival advantage similar to that of MVAC with a better safety profile and tolerability, it is the current standard first-line chemotherapy for advanced/metastatic UC [2, 3]. In a multi-center open-label randomized phase III clinical trial comparing the GC (*n* = 203) and MVAC regimens (*n* = 202), all of the outcomes were similar including OS (HR 1.04, 95% CI 0.82–1.32, *P* = 0.75), PFS (HR 1.05, 95% CI 0.85–1.30), time to treatment failure (HR, 0.89, 95% CI 0.72–1.10), and ORR (GC 49% versus MVAC 46%) [2]. The 5-year OS rates were also similar at 13.0 and 15.3%, respectively (*P *= 0.53) [3]. Regarding treatment-related AE, more GC than MVAC patients had grade 3–4 anemia (27 versus 18%) and thrombocytopenia (57 versus 21%) [2]. On the other hand, more MVAC patients than GC patients had grade 3–4 neutropenia (82 versus 71%), neutropenic fever (14 versus 2%, respectively), neutropenic sepsis (12 versus 1%), grade 3/4 mucositis (22 versus 1%) and alopecia (55 versus 11%). Regarding quality of life (QOL), patients on GC tended to fare better regarding weight, performance status (PS), and fatigue [2].

### Gemcitabine and carboplatin (GCa) regimen

The renal toxicity of platinum-based combinations presents a common problem for patients with metastatic UC. Gemcitabine and carboplatin regimens are used as an option for first-line therapy in cisplatin-ineligible patients with metastatic UC. In a phase II clinical trial for patients with previously untreated advanced UC (*n* = 60) with gemcitabine at a dose of 1000 mg/m^2^ (days 1 and 8) and carboplatin at a dose of area under the concentration curve of 5 (AUC5, day 1), every 21 days for a total of six cycles, intent-to-treat analysis demonstrated an ORR of 38.4% (95% CI 26–51.8%) including 11.7% with CR. The median PFS and OS periods were 7.6 months (95% CI 4.5–10.7 months) and 16.3 months (95% CI 12–20.6 months), respectively [13]. The median OS appeared comparable to that reported for the MVAC and GC combination regimens. Severe (grade 3–4) treatment-related AEs included anemia (18%), thrombocytopenia (23%), and neutropenia (52%) including febrile neutropenia (11%), whereas non-hematologic toxicity was rare [13].

### MVAC regimen

The classic MVAC regimen, which consists of methotrexate, vinblastine, doxorubicin, and cisplatin, was proposed in 1985 by Sternberg et al., and has been used since then to treat metastatic UC [1]. Treatment consists of 4-week (28 days) cycles of 30 mg/m^2^ of methotrexate (day 1), followed by 3 mg/m^2^ of vinblastine, 30 mg/m^2^ of doxorubicin, and 70 mg/m^2^ of cisplatin (day 2), and concluded with repeat vinblastine and methotrexate on days 15 and 22 (Table 1). In this first report, excellent treatment results of tumor regression were noted including 71% ORR and 50% CR [1, 14]. Until its replacement by GC regimen, the MVAC regimen was the standard first-line chemotherapy for a long time; nowadays, it is used as an optional first-line therapy as well as a second-line therapy after the GC regimen.

### High-dose MVAC (HD-MVAC) regimen

To increase the treatment results, a dose-dense schedule of the MVAC regimen has also been proposed. A randomized trial was conducted to evaluate the anti-tumor activity of and survival associated with high-dose-intensity chemotherapy with methotrexate, vinblastine, doxorubicin, and cisplatin (MVAC) plus granulocyte colony-stimulating factor (HD-MVAC) versus MVAC in patients with advanced UC [15]. A total of 263 patients with metastatic or advanced UC who had no prior chemotherapy were randomized to HD-MVAC (2-week cycles) or MVAC (4-week cycles) [15]. Using an intent-to-treat analysis, at a median follow-up of 38 months, on the HD-MVAC arm, there were 62% (95% CI 54–70%) including 21% CRs [15]. On the MVAC arm, there were 50% (95% CI 42–59%) including 9% CRs [15]. The median PFS periods were 9.1 and 8.2 months on the HD-MVAC and MVAC arms, respectively [15]. Although the PFS period was significantly better in the HD-MVAC group (*P *= 0.037, HR 0.75, 95% CI 58–98), there was no statistically significant difference in OS [15]. Nowadays, HD-MVAC is frequently used as neo-adjuvant chemotherapy for patients with locally advanced UC before radical cystectomy or nephroureterectomy.

### Taxane-including regimen

The taxane chemotherapy agents include docetaxel and paclitaxel. Taxane-including regimens have been frequently used as second-line chemotherapy for patients with metastatic UC who progressed after first-line chemotherapy. However, most studies investigating second-line regimens for advanced UC have targeted patients who received MVAC as first-line chemotherapy. Therefore, there is not yet any established regimen to be followed after the failure of GC therapy, which has been widely accepted as a first-line therapy for advanced UC due to its equivalent efficacy and lower toxicity compared to MVAC, which was formerly regarded as the standard first-line chemotherapy prior to the introduction of GC, as described above. Considering the mechanism mediating the acquisition of the resistant phenotype to chemotherapeutic agents, second-line regimens are not likely to include agents integrated into the first-line chemotherapy. Nowadays, most institutions in Japan administer paclitaxel-based chemotherapy (as single agents or combinations) to patients with advanced UC refractory to first-line GC therapy [16]. In a large retrospective study, combination chemotherapy was possibly significantly associated with improved OS compared with single-agent therapy (HR 0.60, *P *= 0.001) [16]. When pembrolizumab therapy is used as a second-line therapy, taxane-including regimens may be used as third-line therapies.

### Paclitaxel/cisplatin/gemcitabin (PCG) regimen

A randomized phase III study compared paclitaxel/cisplatin/gemcitabine (PCG, *n* = 312) and GC (*n* = 314) in patients with metastatic/advanced UC as first-line chemotherapy [17]. Although ORR was better in the PCG arm (55.5%) than in the GC arm (43.6%, *P* = 0.0031), neither the median OS (15.8 months on PCG versus 12.7 months on GC, HR 0.85, *P* = 0.075) nor the PFS period (HR 0.87, *P* = 0.11) was significantly different [17]. Both treatments were well tolerated. However, more thrombocytopenia and bleeding occurred in connection with GC than with PCG (11.4 versus 6.8%, *P* = 0.05), while more febrile neutropenia occurred in connection with PCG than with GC (13.2 versus 4.3%, *P* < 0.001) [17].

### Gemcitabine and paclitaxel (GP) regimen

In a phase II trial designed to compare short-term versus prolonged-term second-line combination chemotherapy of gemcitabine and paclitaxel (GP), neither OS (short-term: 7.8 months, 95% CI 4.2–11.4 months versus prolonged-term: 8.0 months, 95% CI 4.9–11.1 months) nor PFS (short-term: 4.0 months, 95% CI 0–8.0 months versus prolonged-term: 3.1 months, 95% CI 1.9–4.2 months) nor ORR (short-term: 37.5 versus prolonged-term: 41.5%) was significantly different [18]. On prolonged treatment, more patients experienced severe (grade 3–4) anemia (short-term: 6.7% versus prolonged-term: 26.7%, *P* = 0.011) [18]. Although it is not feasible to administer the prolonged regimen, the high ORR (around 40%) of GP is considered to prove that it is a promising second-line treatment option for patients with metastatic UC [18].

### Ramucirumab and docetaxel

Ramucirumab (Cyramza^®^, Eli Lilly) is a human IgG1 monoclonal antibody against vascular endothelial growth factor receptor (VEGFR)-2. A ramucirumab plus docetaxel regimen demonstrated superior PFS period over chemotherapy in patients with platinum-refractory metastatic UC [19]. This evidence validates the inhibition of VEGFR-2 signaling as a potential new therapeutic treatment option for patients with UC. In a randomized double-blind, phase III trial in patients with metastatic UC who had progressed during or after platinum-based chemotherapy, patients received intravenous docetaxel 75 mg/m^2^ plus either intravenous ramucirumab 10 mg/kg (*n* = 263) or matching placebo (*n* = 267) on day 1 of each repeating 21-day cycle [19]. The PFS period was prolonged significantly in patients treated with ramucirumab plus docetaxel versus placebo plus docetaxel (median 4.07 versus 2.76 months, HR 0.757, *P *= 0.0118) [18]. ORR was achieved by 24.5% (95% CI 18.8–30.3%) and 14.0% (95% CI 9.4–18.6%) of patients treated with ramucirumab and placebo, respectively [19]. Regarding treatment-related AEs, the frequency of severe (grade 3–4) AEs was similar in patients treated with ramucirumab and placebo (60 versus 62%) [19]. Ramucirumab has not yet been approved in Japan for the treatment of metastatic UC. Eventually, however, as these positive results are likely to permit its approval, the combination of ramucirumab and docetaxel will become an important option for second-line as well as third-line therapy after pembrolizumab.

### Vinflunine

Vinflunine is a microtubule inhibitor that is effective for patients with metastatic UC although this agent has not yet been approved in Japan. A randomized phase III study compared vinflunine (*n* = 253, PS = 0: 320 mg/m^2^, every 3 weeks; PS = 0 with previous pelvic radiation and PS = 1: 280 mg/m^2^ subsequently escalated to 320 mg/m^2^) in combination with best supportive care (BSC) and BSC (*n* = 117) alone in the treatment of patients with metastatic UC who had experienced progression after a first-line platinum-containing regimen [20]. In the intent-to-treat population, the objective of a median 2-month survival advantage (6.9 months for vinflunine plus BSC versus 4.6 months for BSC alone) was achieved (HR, 0.88, 95% CI 0.69–1.12) but was not statistically significant (*P* = 0.287) [20]. However, multivariate Cox analysis adjusting for prognostic factors showed a statistically significant effect of vinflunine on OS (*P* = 0.036, HR 0.77, 95% CI 0.61–0.98) [20]. In the eligible population (*n* = 357), the median OS was also significantly longer for vinflunine plus BSC than it was for BSC alone (6.9 versus 4.3 months), with the difference being statistically significant (*P* = 0.040) [20].

### Biomarkers

Among the biomarkers for metastatic urothelial cancer, Bajorin score, which incorporates Karnofsky performance status (KPS) less than 80% and presence of visceral (lung, liver, or bone) metastasis, is the best known and most frequently used stratification factor in the clinical trials [21]. In a phase II study of patients with metastatic UC (*n* = 203) who were undergoing MVAC chemotherapy, predictive prognostic factors were retrospectively analyzed by multivariate regression analysis [21]. Two factors were extracted as independent indicators of poor prognosis: KPS less than 80% and the presence of visceral metastasis [21]. Median survival times for patients who had zero, one, and two risk factors were 33, 13.4, and 9.3 months, respectively (*P* = 0.0001) [21].

Regarding the view from the genetic factors, expression level of various drug resistance and susceptible genes have been disclosed to associate with efficacy of cytotoxic chemotherapy [22–24]. Excision repair cross complementing 1 (ERCC1) is the nucleotide excision repair enzyme, which is involved in cisplatin-resistance [22]. Ribonucleotide reductase subunit M1 (RRM1) functions DNA repair after chemotherapy damage [22, 23]. These chemotherapy resistance genes are considered to inhibit the efficacy of cytotoxic chemotherapy for the patients with metastatic UC [22, 23]. On the other hand, the human equilibrative nucleoside transporter 1 (hENT1) functions major nucleoside transporter and facilitates efficient delivery of gemcitabine into cancer cells [24]. Hence, it might increase the efficacy of the combination of gemcitabine and platinum-based chemotherapy [24]. Various genes including these genes should be associated with the efficacy of medical therapy. Recent advances of precision medicine using the next generation sequencer may shed light to predict the response and prognosis. Furthermore, as described above, on current, various clinical trials, which is comparing immune-checkpoint inhibitors monotherapy and combination of immune-checkpoint inhibitors with standard chemotherapy as first-line therapy for patients with advanced UC. Expression level of these drug function and/or resistance associated genes might become one of the key factors whether we decide to undergo checkpoint inhibitors monotherapy or combination therapy with cytotoxic chemotherapy.

At the same time, there are several other biomarkers for immune-checkpoint inhibitor therapy, as we introduced very recently [25]. Based on the mechanism of efficacy, the number of neoantigens and expression of MHC molecules are strong candidate biomarkers [25]. Despite the various interference factors, which include antibody used, immunohistochemical procedure, cut-off point of stained sample, newly corrected specimen versus archival tumor sample, heterogeneity between primary and metastatic sites, and heterogeneity among metastatic sites, PD-1/PD-L1 expression can be considered a potential biomarker [25]. As more treatment options become available, more biomarkers need to be established.

## Conclusion

In this review, we introduced the current US-FDA-approved immune-checkpoint inhibitors, including pembrolizumab, which has just become available for clinical practice in the treatment of metastatic UC in Japan. Based on its promising anti-tumor efficacy and manageable safety profile as demonstrated in the phase III KEYNOTE-045 trial, pembrolizumab therapy is expected to be rapidly introduced in clinical practice for metastatic UC in Japan. In addition, we summarized the cytotoxic chemotherapies as they still represent the mainstay of first-line therapy as well as useful options for second or later lines. The options proposed for current (2018) possible medical therapy for patients with metastatic UC in Japan are listed in Table 3. At this time, various combination therapies including various combinations of immune-checkpoint inhibitors with cytotoxic chemotherapy and combinations of double immune-checkpoint inhibitors are in clinical trials. We await the results of these trials with high hopes for new therapies.

**Table 3:** Proposed medical therapies for treatment of patients with metastatic urothelial cancer in Japan (2018)

	First-line therapy	Second-line therapy
Cisplatin-eligible		
Standard	GC (gemcitabine and cisplatin)	Pembrolizumab
Option	MVAC (methotrexate, vinblastine, adriamycin, cisplatin)	Taxane-including regimen such as PCG (paclitaxel, cisplatin, gemcitabine)
Cisplatin-ineligible		
Standard	GCa (gemcitabine and carboplatin)	Pembrolizumab
Option		Taxane-including regimen such as GP (gemcitabine and paclitaxel)

## References

[1] Sternberg CN, Yagoda A, Scher HI (1985). Preliminary results of M-VAC (methotrexate, vinblastine, doxorubicin and cisplatin) for transitional cell carcinoma of the urothelium. J Urol.

[2] von der Maase H, Hansen SW, Roberts JT (2000). Gemcitabine and cisplatin versus methotrexate, vinblastine, doxorubicin, and cisplatin in advanced or metastatic bladder cancer: results of a large, randomized, multinational, multicenter, phase III study. J Clin Oncol.

[3] von der Maase H, Sengelov L, Roberts JT (2005). Long-term survival results of a randomized trial comparing gemcitabine plus cisplatin, with methotrexate, vinblastine, doxorubicin, plus cisplatin in patients with bladder cancer. J Clin Oncol.

[4] Dudley JC, Lin MT, Le DT (2016). Microsatellite instability as a biomarker for PD-1 blockade. Clin Cancer Res.

[5] Schumacher TN, Schreiber RD (2015). Neoantigens in cancer immunotherapy. Science.

[6] Bellmunt J, de Wit R, Vaughn DJ (2017). Pembrolizumab as second-line therapy for advanced urothelial carcinoma. N Engl J Med.

[7] Balar AV, Castellano D, O’Donnell PH (2017). First-line pembrolizumab in cisplatin-ineligible patients with locally advanced and unresectable or metastatic urothelial cancer (KEYNOTE-052): a multicentre, single-arm, phase 2 study. Lancet Oncol.

[8] Motzer RJ, Escudier B, McDermott DF (2015). Nivolumab versus everolimus in advanced renal-cell carcinoma. N Engl J Med.

[9] Sharma P, Retz M, Siefker-Radtke A (2017). Nivolumab in metastatic urothelial carcinoma after platinum therapy (CheckMate 275): a multicentre, single-arm, phase 2 trial. Lancet Oncol.

[10] Powles T, Durán I, van der Heijden MS, et al (2017) Atezolizumab versus chemotherapy in patients with platinum-treated locally advanced or metastatic urothelial carcinoma (IMvigor211): a multicentre, open-label, phase 3 randomised controlled trial. Lancet. 10.1016/s0140-6736(17)33297-x 10.1016/S0140-6736(17)33297-X 29268948

[11] Powles T, O’Donnell PH, Massard C (2017). Efficacy and safety of durvalumab in locally advanced or metastatic urothelial carcinoma: updated results from a phase 1/2 open-label study. JAMA Oncol.

[12] Patel MR, Ellerton J, Infante JR (2017). Avelumab in metastatic urothelial carcinoma after platinum failure (JAVELIN Solid Tumor): pooled results from two expansion cohorts of an open-label, phase 1 trial. Lancet Oncol.

[13] Bamias A, Moulopoulos LA, Koutras A (2006). The combination of gemcitabine and carboplatin as first-line treatment in patients with advanced urothelial carcinoma. A Phase II study of the Hellenic Cooperative Oncology Group. Cancer.

[14] Sternberg CN, Yagoda A, Scher HI (1989). Methotrexate, vinblastine, doxorubicin, and cisplatin for advanced transitional cell carcinoma of the urothelium. Efficacy and patterns of response and relapse. Cancer.

[15] Sternberg CN, de Mulder PH, Schornagel JH (2001). European Organization for Research and Treatment of Cancer Genitourinary Tract Cancer Cooperative Group. Randomized phase III trial of high-dose-intensity methotrexate, vinblastine, doxorubicin, and cisplatin (MVAC) chemotherapy and recombinant human granulocyte colony-stimulating factor versus classic MVAC in advanced urothelial tract tumors: European Organization for Research and Treatment of Cancer Protocol no. 30924. J Clin Oncol.

[16] Sonpavde G, Pond GR, Choueiri TK (2016). Single-agent taxane versus taxane-containing combination chemotherapy as salvage therapy for advanced urothelial carcinoma. Eur Urol.

[17] Bellmunt J, von der Maase H, Mead GM (2011). Randomized phase III study comparing paclitaxel/cisplatin/gemcitabine and gemcitabine/cisplatin in patients with locally advanced or metastatic urothelial cancer without prior systemic therapy: EORTC Intergroup Study 30987. J Clin Oncol.

[18] Albers P, Park SI, Niegisch G (2011). Randomized phase III trial of 2nd line gemcitabine and paclitaxel chemotherapy in patients with advanced bladder cancer: short-term versus prolonged treatment [German Association of Urological Oncology (AUO) trial AB 20/99]. Ann Oncol.

[19] Petrylak DP, de Wit R, Chi KN (2017). Ramucirumab plus docetaxel versus placebo plus docetaxel in patients with locally advanced or metastatic urothelial carcinoma after platinum-based therapy (RANGE): a randomised, double-blind, phase 3 trial. Lancet.

[20] Bellmunt J, Théodore C, Demkov T (2009). Phase III trial of vinflunine plus best supportive care compared with best supportive care alone after a platinum-containing regimen in patients with advanced transitional cell carcinoma of the urothelial tract. J Clin Oncol.

[21] Bajorin DF, Dodd PM, Mazumdar M (1999). Long-term survival in metastatic transitional-cell carcinoma and prognostic factors predicting outcome of therapy. J Clin Oncol.

[22] Bellmunt J, Paz-Ares L, Cuello M (2007). Gene expression of ERCC1 as a novel prognostic marker in advanced bladder cancer patients receiving cisplatin-based chemotherapy. Ann Oncol.

[23] Matsumura N, Nakamura Y, Kohjimoto Y (2017). Overexpression of ribonucleotide reductase subunit M1 protein predicts shorter survival in metastatic bladder cancer patients treated with gemcitabine-containing combination chemotherapy. Int J Urol.

[24] Matsumura N, Nakamura Y, Kohjimoto Y (2011). The prognostic significance of human equilibrative nucleoside transporter 1 expression in patients with metastatic bladder cancer treated with gemcitabine-cisplatin-based combination chemotherapy. BJU Int.

[25] Yuasa T, Masuda H, Yamamoto S (2017). Biomarkers to predict prognosis and response to checkpoint inhibitors. Int J Clin Oncol.

